# Historical Contingencies Modulate the Adaptability of *Rice Yellow Mottle Virus*

**DOI:** 10.1371/journal.ppat.1002482

**Published:** 2012-01-26

**Authors:** Nils Poulicard, Agnès Pinel-Galzi, Oumar Traoré, Florence Vignols, Alain Ghesquière, Gnissa Konaté, Eugénie Hébrard, Denis Fargette

**Affiliations:** 1 Institut de Recherche pour le Développement (IRD), UMR RPB (IRD, CIRAD, Université Montpellier 2), Montpellier, France; 2 Institut de l'Environnement et de Recherches Agricoles (INERA), Ouagadougou, Burkina-Faso; 3 Institut de Recherche pour la Développement (IRD), UMR DIADE (IRD, CIRAD, Université Montpellier 2), Montpellier, France; Pasteur Institute, France

## Abstract

The *rymv1-2* and *rymv1-3* alleles of the *RYMV1* resistance to *Rice yellow mottle virus* (RYMV), coded by an *eIF(iso)4G1* gene, occur in a few cultivars of the Asiatic (*Oryza sativa*) and African (*O. glaberrima*) rice species, respectively. The most salient feature of the resistance breaking (RB) process is the converse genetic barrier to *rymv1-2* and *rymv1-3* resistance breakdown. This specificity is modulated by the amino acid (glutamic acid vs. threonine) at codon 49 of the Viral Protein genome-linked (VPg), a position which is adjacent to the virulence codons 48 and 52. Isolates with a glutamic acid (E) do not overcome *rymv1-3* whereas those with a threonine (T) rarely overcome *rymv1-2*. We found that isolates with T49 had a strong selective advantage over isolates with E49 in *O. glaberrima* susceptible cultivars. This explains the fixation of the mutation T49 during RYMV evolution and accounts for the diversifying selection estimated at codon 49. Better adapted to *O. glaberrima*, isolates with T49 are also more prone than isolates with E49 to fix *rymv1-3* RB mutations at codon 52 in resistant *O. glaberrima* cultivars. However, subsequent genetic constraints impaired the ability of isolates with T49 to fix *rymv1-2* RB mutations at codons 48 and 52 in resistant *O. sativa* cultivars. The origin and role of the amino acid at codon 49 of the VPg exemplifies the importance of historical contingencies in the ability of RYMV to overcome *RYMV1* resistance.

## Author Summary

A fundamental question in virus evolution is whether emergence into a new host species/cultivar requires adaptation of the virus during the early stages of infection or whether it is largely via a chance transmission of a viral strain with the requisite characteristics. Our studies of the breakdown of *RYMV1* resistance to *Rice yellow mottle virus* (RYMV) in rice show that emergence of a virus into a new host is a two-step process, where adaptive mutations can be fixed only by isolates with the necessary genetic determinants. We found the identity, effects and origin of these genetic determinants. Past host adaptation and subsequent genetic constraints, epitomized in a single amino acid, modulate the current ability of RYMV to overcome *RYMV1* resistance through epistatic relationships. The importance of epistatic interactions in virus adaptation is increasingly acknowledged, but has seldom been studied within an historical context. The origin and role of the genetic determinants in *RYMV1* resistance breakdown exemplify the importance of historical contingencies in the adaptability of a virus to a new host, a scenario at variance with the widely accepted co-evolution arm race model between pathogens and their hosts.

## Introduction

*Rice yellow mottle virus*, of the genus *Sobemovirus*, causes a major disease in Africa [1]. The virus has a narrow host range restricted to the two cultivated rice species, *Oryza sativa* and *O. glaberrima*, and a few wild grasses [2]. The Asiatic rice *O. sativa*, which was introduced to East Africa and Madagascar in the 11^th^ century and to West Africa in the 15^th^ century, is now grown throughout Africa. In contrast, the African rice *O. glaberrima*, which was domesticated in the Niger Interior Delta ca. 3000 years ago, has been grown in West Africa only [3]–[6]. A few rice cultivars exhibit a high resistance to RYMV characterized by an absence of symptoms and no viral detection by ELISA [7]. The inheritance of this high resistance is recessive. The resistance gene *RYMV1* was identified as an *eIF(iso)4G* gene [8]. Four *rymv1* resistance alleles have been characterized, one in *O. sativa* (*rymv1-2*) and three in *O. glaberrima* cultivars (*rymv1-3*, *rymv1-4, rymv1-5*) [9]. The genetic determinants of the ability to break resistance alleles *rymv1-2*, *rymv1-3* and *rymv1-4* were investigated in this study.

**Figure 1:**
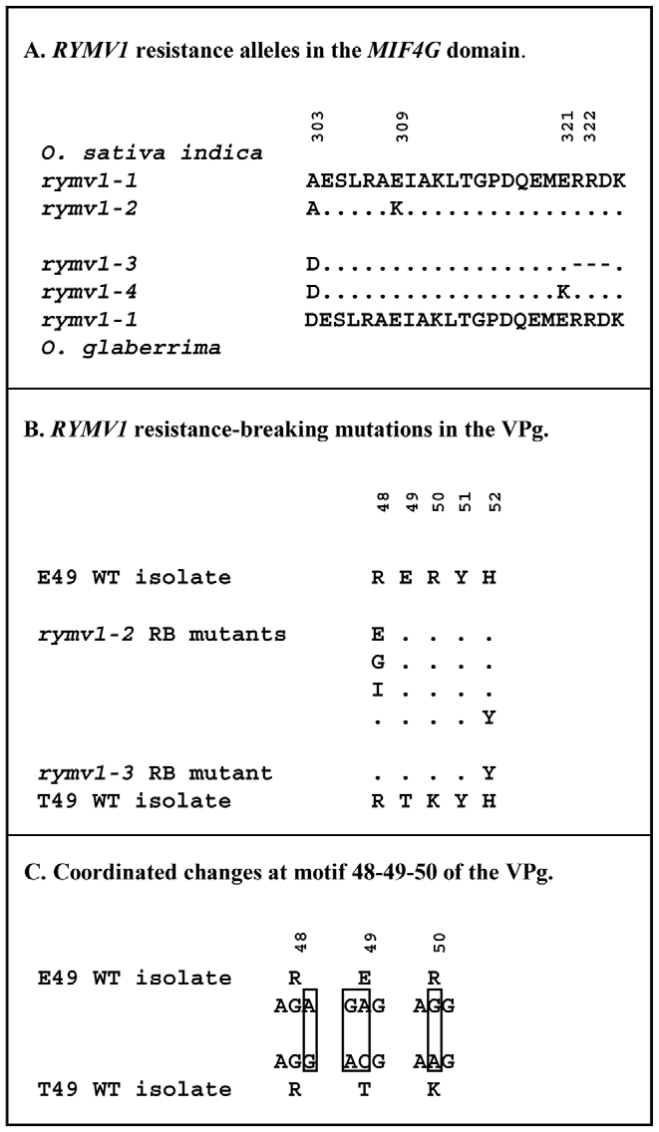
Genetic characteristics of the plant and virus material used in the study. (A) *RYMV1* resistance alleles in the MIF4G domain. Alignment of the 303-325 region of the RYMV1 protein (part of the conserved MIF4G domain) from susceptible (*rymv1-1*) and resistant *O. sativa* (*rymv1-2*) and *O. glaberrima* (*rymv1-3* and *rymv1-4*) accessions. Position 303 distinguishes *O. sativa* from *O. glaberrima* species; adapted from [9]. (B) *RYMV1* resistance-breaking mutations in the VPg. Alignment of the 48-52 region of the central part of the VPg from the wild-type (WT) isolates with E49 and T49 and from the main *rymv1-2* and *rymv1-3* resistance breaking (RB) mutants; adapted from [13], [15]. (C) Coordinated changes at codons 48-49-50 of the VPg. Coordinated changes (boxed) at codons 48, 49 and 50 in the central domain of the VPg of WT isolates with E49 and T49; adapted from [13].

The genetic determinants of *RYMV1* resistance are located in the central domain of eIF(iso)4G1 (named MIF4G domain) [8], [10]. The resistance allele *rymv1-2* of the two *O. sativa* cultivars Gigante and Bekarosaka is due to a point mutation leading to the replacement of a glutamic acid by a lysine at codon 309 (Figure 1a). The resistance allele *rymv1-3* of the cultivar Tog5681 and of a few other *O. glaberrima* cultivars [9], [11] is caused by a deletion of codons 322-324 [8]. The *rymv1-4* resistance allele of the cultivar Tog5672 and of a few other *O. glaberrima* cultivars is due to a point mutation resulting in the replacement of a glutamic acid by a lysine at codon 321 [8]. Interestingly, there is a single amino acid-specific difference between *O. glaberrima* and *O. sativa* at position 303 of the MIF4G domain, close to the genetic determinants of *RYMV1* resistance. An alanine occurs in *O. sativa* whereas an aspartic acid exists in *O. glaberrima* (Figure 1a). This result was first established on a few sequences [8], [9] and recently confirmed on a larger data set (C. Lirette and E. Hébrard, unpublished results).

Earlier studies established the features of the *RYMV1* resistance breakdown (RB) process [12]–[15]. In most instances, the inoculation of resistant plants does not cause infection. Occasionally however, fully systemic symptoms are expressed, the virus content reaches that of susceptible plants, and the virus is readily transmitted from plant-to-plant: i.e. the resistance is overcome. Subsequently, wild-type (WT) isolates are displaced in the resistant plants by newly-adapted mutants with higher multiplication rates. Resistance-breaking process involves the Viral Protein genome linked (VPg), which is an intrinsically disordered protein [16]. The RB mutations occur in codons - referred to as virulence codons - in the central domain of the VPg between positions 37 and 52. The *rymv1-2* resistance is most frequently overcome by the substitution of arginine (R) by glycine (G) at codon 48 and then of glycine by glutamic acid (E) at the same codon [13] (Figure 1b). Minor RB mutations occur at codons 48 (R48I and R48W), 42 (N42Y), 43 (T43A) and 52 (H52Y). The *rymv1-3* resistance breakdown of the *O. glaberrima* cv. Tog5681 involves the replacement of serine (S) by proline (P) or alanine (A) at codon 41, and/or the replacement of histidine (H) by tyrosine (Y) at codon 52 [15]. Interestingly, H52Y is the only RB mutation that overcomes both *rymv1-2* and *rymv1-3* resistance alleles. Recently, the direct interaction between the MIF4G domain and the VPg was shown by co-purification and two-hybrid assays [17]. Mutations mimicking the resistance alleles in MIF4G strongly diminished the interaction with the VPg. Virulence mutations that become fixed in the VPg partially or fully restored the interaction.

One of the most salient feature of *RYMV1* RB process is the converse genetic barrier to *rymv1-2* and *rymv1-3* resistance breakdown. Isolates that can break *rymv1-2* never overcome *rymv1-3* whereas isolates that can break *rymv1-3* rarely overcome *rymv1-2*. This specificity is associated with the amino acid at codon 49 of the VPg, a position which is adjacent to the main virulence codons 48 and 52. Isolates with a glutamic acid (E) do not overcome *rymv1-3* [15] whereas those with a threonine (T) rarely overcome *rymv1-2* [13]. The changes at codon 49 are coordinated with those at the adjacent codons 48 and 50 (Figure 1c). Mutations R(AGA)48R(AGG), E(GAG)49T(ACG) and R(AGG)50K(AAG) are generally associated, R(AGA)48E49R50 being the ancestral motif and R(AGG)48T49K50 being the derived motif [13]. There are, however, a few isolates with the REK motif [13]. This stretch of four coordinated changes (one synonymous and three non-synonymous) at codons 48-49-50 is a notable case of epistasis [18].

In this study, the identity, role and origin of the genetic determinants of the ability to break *RYMV1* resistance alleles were investigated. First, we tested whether the amino acid at codon 49 was a key genetic determinant and not just a molecular ‘signature’ of the ability to overcome the *RYMV1* resistance alleles. Second, we examined the effects of the amino acid at codon 49 on the fixation of RB mutations at the adjacent virulence codons 48 and 52. Third, we studied whether the threonine and glutamic acid at codon 49 resulted from past adaptation to *O. glaberrima* and *O. sativa*, respectively. This hypothesis arose from the matching geographical distribution of the amino acid polymorphism at codon 49 of the VPg of the virus and that of the two cultivated rice species in Africa. *O. glaberrima* cultivars and RYMV isolates with T49 occur in West Africa only, whereas *O. sativa* cultivars and RYMV isolates with E49 occur everywhere in Africa [3], [15]. Moreover, codon 49 is under strong diversifying selection [13], which may reflect past adaptation to rice species. In conclusion, we found that RYMV-rice relationships do not fit a simple co-evolutionary arms race model. An alternative evolutionary scenario is proposed that emphasizes the role of historical contingencies in virus adaptation to a new host.

## Results

### The amino acid at codon 49 of the VPg is a key genetic determinant of the ability to break *RYMV1* resistance

The mutant CIa*49E was obtained from the infectious clone of the isolate CIa (T49) to establish the causal role of the amino acid at codon 49 in the ability to break *RYMV1* resistance alleles. The RB ability of the isolate CIa (T49) and of the mutant CIa*49E were then compared. The isolate CIa and the mutant CIa*49E were inoculated at the same concentration to the *rymv1-2* resistant *O. sativa indica* cv. Gigante and to the *rymv1-3* resistant *O. glaberrima* cv. Tog5681. Forty-two days after the inoculation of the isolate CIa (T49), ca. 96% of the *rymv1-3* resistant plants but only ca. 5% of the *rymv1-2* resistant plants showed severe symptoms and reached a high virus content (Table 1). The high ability of the isolate CIa (T49) to overcome *rymv1-3* and its low ability to overcome *rymv1-2* are typical of isolates with T49 [13], [15]. The substitution of threonine by a glutamic acid at codon 49 of the mutant CIa*49E reversed these trends. The mutant CIa*49E fully lost its ability to overcome *rymv1-3* whereas its ability to overcome *rymv1-2* increased dramatically from 5 to 40%. Consequently, the resistance breaking pattern of the mutant CIa*49E became similar to that of isolates with E49. The link between the amino acid at codon 49 and the ability to break the *rymv1-2* and *rymv1-3* resistance alleles was highly significant (χ^2^ = 17.5, *P*<0.001 and χ^2^ = 95.3, *P*<0.001 for *rymv1-2* and *rymv1-3*, respectively).

**Table 1:** Breakdown of *rymv1-2* and *rymv1-3* rice resistant cultivars after inoculation of the RYMV isolate CIa (T49) and of the mutant CIa*49E.

Virus isolate	Rice species			
	*O. sativa indica*		*O. glaberrima*	
	cv. Gigante	cv. IR64	cv. Tog5681	cv. Tog5673
	*rymv1-2* 1	*rymv1-1* 2	*rymv1-3*	*rymv1-1* 2
CIa (T49)	3/53 (5%)	10/10 (100%)	51/53 (96%)	10/10 (100%)
CIa*49E	20/50 (40%)	10/10 (100%)	0/50 (0%)	10/10 (100%)

1 ratio of the number of plants infected, assessed by DAS-ELISA 42 days post inoculation, over the number inoculated, and percentage into brackets.

2 susceptible control.

Not only did the substitution of threonine by glutamic acid at codon 49 in the mutant CIa*49E reverse the *RYMV1* RB ability of isolate CIa (T49), but it also modified the mutational pathways. Sequencing the viral population after *rymv1-2* resistance breakdown by the mutant CIa*49E revealed the presence of the *48G mutation (i.e. the first step of the major mutational pathway toward *rymv1-2* resistance breakdown). Furthermore, minor *rymv1-2* RB mutations *42Y and *43A were observed. These mutations are sometimes detected after *rymv1-2* resistance breakdown by isolates with E49 but never by isolates with T49 [13], [15]. Moreover, the *rymv1-2* RB mutation *48W which sometimes emerged after the inoculation of the isolates with T49 [13], [15] was not detected after the inoculation of the mutant CIa*49E. Considering both its resistance breaking ability and the kind of RB mutations fixed, the mutant CIa*49E responded like the isolates with E49, and no longer like the isolate CIa (T49) or any other isolates with T49. Overall, these results show that the amino acid at codon 49 is a key genetic determinant of the ability to overcome *rymv1-2* and *rymv1-3* resistance alleles.

### Effect of a threonine at codon 49 on the fixation of *rymv1-2* RB mutations at codon 48

**Figure 2:**
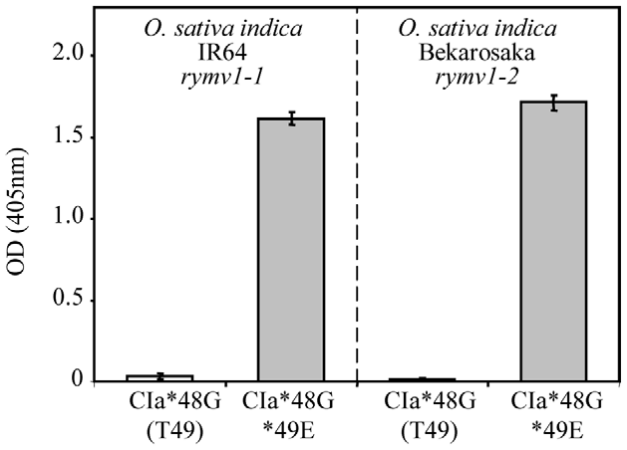
Effect of the amino acid at codon 49 on accumulation of the *rymv1-2* RB mutants with *48G. Virus content of the double mutant CIa*48G*49E (grey histograms) and of the single mutant CIa*48G (T49) (white histograms) in the *rymv1-1* susceptible *O. sativa indica* cv. IR64 and in the *rymv1-2* resistant *O. sativa* cv. Bekarosaka assessed by DAS-ELISA (absorbance at 405 nm). The vertical bars show the standard deviation of the mean calculated from five plants.

It has been established that directed mutagenesis resulting in the replacement of arginine at codon 48 of the VPg by glycine within the infectious clone CIa (T49) was lethal to the virus [13]. It was then postulated that it was the cause of the inability of isolates with T49 to follow the main *rymv1-2* RB mutational pathway R>G>E at codon 48. To test this hypothesis, the double mutant CIa*48G*49E was constructed by directed mutagenesis, and its infectivity was compared with that of the single mutant CIa*48G (T49). The viral RNAs obtained by *in vitro* transcription were inoculated to the susceptible *O. sativa indica* cv. IR64 and to the *rymv1-2* resistant cv. Bekarosaka. Thirty days after inoculation, the single mutant CIa*48G (T49) was not detected by DAS-ELISA either in the susceptible or the resistant cultivar (Figure 2), confirming that the combination of amino acids G48-T49 was unfit. By contrast, all plants - either resistant or susceptible - inoculated with the double mutant CIa*48G*49E expressed symptoms in non-inoculated leaves and reached a high virus content.

**Figure 3:**
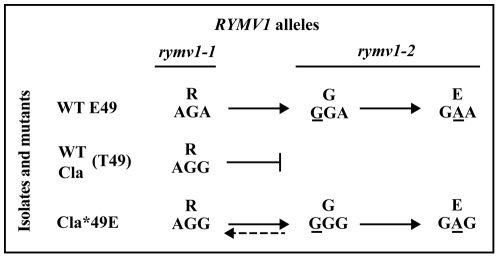
Effect of the amino acid at codon 49 on the *rymv1-2* mutational pathway. Wild-type (WT) isolates with E49 follow the two-step major mutational pathway R>G>E at codon 48, whereas WT isolates with T49, including isolate CIa, are unable to do so (adapted from [13]. Mutation *49E in the isolate CIa restored the ability of the mutant to follow the *rymv1-2* major mutational pathway at codon 48, subsequently responding like isolates with E49. The dotted arrow indicates a reversion to the wild type.

The infection of the *rymv1-2* resistant plants by the mutant CIa*49E (R48) was followed. As mentioned above, the *48G mutation was fixed after the inoculation of the mutant CIa*49E (R48) to the *rymv1-2* resistant plants. At 90 days post-inoculation (dpi), the mutant CIa*48G*49E was itself displaced by CIa*48E*49E (Figure 3). Interestingly, synonymous differences at codon 48 R(AGG or AGA), G(GGG or GGA) and E(GAG or GAA) did not interfere with the ability of the mutant CIa*49E to follow the mutational pathway R>G>E. Reversion from CIa*48E*49E to CIa*48G*49E in susceptible rice cultivars has been reported earlier [14]. In the present experiment, reversion from CIa*48G*49E to CIa*48R*49E was observed in susceptible cultivars. Altogether, the substitution of threonine by glutamic acid at codon 49 of the VPg of isolate CIa restored the viability of the mutant CIa*48G*49E and also its ability to adapt to *rymv1-2* resistant cultivars or to revert to the avirulent form in susceptible hosts. The mutant CIa*49E responded like the isolates with E49 and no longer like the isolate CIa (T49) or any other isolates with T49. These results validated the claim that lack of fitness of genotypes with the combination G48-T49 is the cause of the inability of isolates with T49 to overcome *rymv1-2* by the main mutational pathway R>G>E at codon 48. Collectively, these results demonstrate the critical effect of the amino acid at codon 49 on the ability to fix *rymv1-2* RB mutations at the virulence codon 48.

### Effect of a glutamic acid at codon 49 on the fixation of *rymv1-3* RB mutations at codon 52

Mutation H52Y is unique in that it breaks both *rymv1-2* and *rymv1-3* resistance alleles. However, the fixation of mutation *52Y depends upon the amino acid at codon 49. In *rymv1-2* resistance-breakdown, the mutation *52Y is only fixed after inoculation of isolates with E49 [13] whereas for the *rymv1-3* resistant cv. Tog5681, the mutation *52Y is only fixed after the inoculation of isolates with T49 [15]. Several isolates with *52Y and T49 (CIa, Ma105 and Ma203) or E49 (Mg16) obtained earlier during passage experiments [13], [15] were inoculated to *rymv1-2* resistant *O. sativa indica* cvs. Gigante and Bekarosaka and to *rymv1-3* resistant *O. glaberrima* cv. Tog5681. The results depended on the amino acid at codon 49. The isolates with T49 and *52Y were infectious in both *rymv1-2* and *rymv1-3* resistant plants, whereas the isolates with E49 and *52Y were infectious in *rymv1-2* but not in *rymv1-3* resistant plants (Table 2).

**Table 2:** Infectivity of RYMV isolates with T49 or E49 and RB mutation *52Y after inoculation of *rymv1-2* and *rymv1-3* rice resistant cultivars.

RB genotypes	Rice species	
	*O. sativa indica*	*O. glaberrima*
	cvs. Gigante/Bekarosaka	cv. Tog5681
	*rymv1-2*	*rymv1-3*
2CIa*52Y (T49)	6/28 (21%)^1^	5/5 (100%)
2Ma105*52Y (T49)	20/20 (100%)	5/5 (100%)
2Ma203*52Y (T49)	20/20 (100%)	5/5 (100%)
3Mg16*52Y (E49)	5/5 (100%)	0/20 (0%)
4CIa*49E*52Y	9/9 (100%)	0/35 (0%)

1 ratio of the number of plants infected over the number inoculated; percentage into brackets.

2 RB genotypes obtained after inoculation of WT isolates to *rymv1-3* resistant plants.

3 RB genotype obtained after inoculation of the WT isolates to *rymv1-2* resistant plants.

4 RB genotype constructed by directed mutagenesis of the infectious clone CIa.

**Figure 4:**
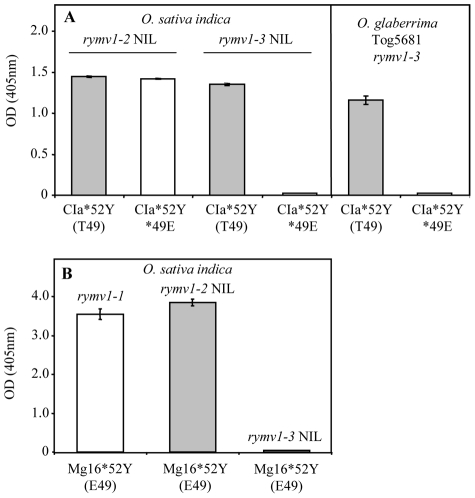
Effect of the amino acid at codon 49 on virus accumulation in *RYMV1* resistant plants. (A) Virus accumulation of the single mutant CIa*52Y (T49) (grey bars) and of the double mutant CIa*49E*52Y (white bars) in the *rymv1-2* and in the *rymv1-3* resistant NILs, and in the *rymv1-3* resistant *O. glaberrima* cv. Tog5681 assessed by DAS-ELISA (absorbance at 405 nm). The vertical bars show the standard deviation of the mean calculated from 25 plants. (B) Virus accumulation of the single mutant Mg16*52Y (E49) in the *rymv1-1* susceptible *O. sativa indica* cv. IR64 (white histogram), in the *rymv1-2* resistant NIL (grey histogram) and in the *rymv1-3* resistant NIL assessed by DAS-ELISA (absorbance at 405 nm). The vertical bars show the standard deviation of the mean calculated from 25 plants.

The single mutant CIa*52Y (T49) and the double mutant CIa*49E*52Y were constructed to establish the role of the amino acid at codon 49 in this contrasted infectivity. The mutants were then inoculated at the same concentration to the *rymv1-2* resistant cv. Bekarosaka and to the *rymv1-3* resistant cv. Tog5681. The single mutant CIa*52Y (T49) was detected at a high concentration in both resistant cultivars. In contrast, the double mutant CIa*49E*52Y was infectious in cv. Bekarosaka but not in cv. Tog5681 (Table 2). Two nearly isogenic lines (NILs) were tested to understand the role of the rice genetic background on the relationship between codons 49 and 52 during the *RYMV1* RB process. The viral accumulation of the single mutant CIa*52Y (T49) and that of the double mutant CIa*49E*52Y in each NIL and in the parental *O. glaberrima* resistant cv. Tog5681 was compared. At 30 dpi, the single mutant CIa*52Y (T49) genotype was detected at high levels in both *rymv1-2* and *rymv1-3* resistant plants (Figure 4a). In contrast, the double mutant CIa*49E*52Y was infectious in the *rymv1-2* NIL, but not in the resistant *O. glaberrima* cv. Tog5681 or in the *rymv1-3* NIL. Similarly, the isolate Mg16*52Y, an RB isolate with E49 of a different strain, was not infectious in *rymv1-3* resistant cultivar cv. Tog5681 and NILs (Figure 4b). Thus, the presence of a threonine at codon 49 is a prerequisite for fixation of RB mutation *52Y in *rymv1-3* resistant plants. Conversely, isolates with E49 are unable to fix the *rymv1-3* RB mutation *52Y.

It was earlier established that *rymv1-2* RB mutants with E48 or Y52 were not able to overcome *rymv1-4* although a similar substitution - glutamic acid into a lysine - causes the *rymv1-2* and *rymv1-4* resistance at positions 309 and 321, respectively [12]. In contrast, the response to RYMV inoculation of *rymv1-3* and *rymv1-4* resistance alleles, which both occur in *O. glaberrima* cultivars, showed marked similarities: (i) the single mutant CIa*52Y (T49) was infectious in cv. Tog5672, although at a lower rate than in the *rymv1-3* resistant cv. Tog5681; (ii) only isolates with T49 were able to break the *rymv1-4* resistance; (iii) after inoculation of isolate Ng25 (T49), the RB mutation S41P was fixed to *rymv1-3* resistant cv. Tog5681 and *rymv1-4* resistant cv. Tog5672 (data not shown). These similarities in the *rymv1-4* and *rymv1-3* in the RB process suggest that the breakdown of *RYMV1* resistance is host-species dependent. Comparison of the response of the parental lines and NILs indicates that the origin of the contrasted responses between *rymv1-2* and *rymv1-3* occurs in the MIF4G domain rather than elsewhere in the genome. Position 303 is the most likely candidate as it is the only position that distinguishes *O. sativa* from *O. glaberrima* and it is located close to the genetic determinants of resistance.

### Effect of the amino acid at codon 49 on the VPg/MIF4G interaction

**Figure 5:**
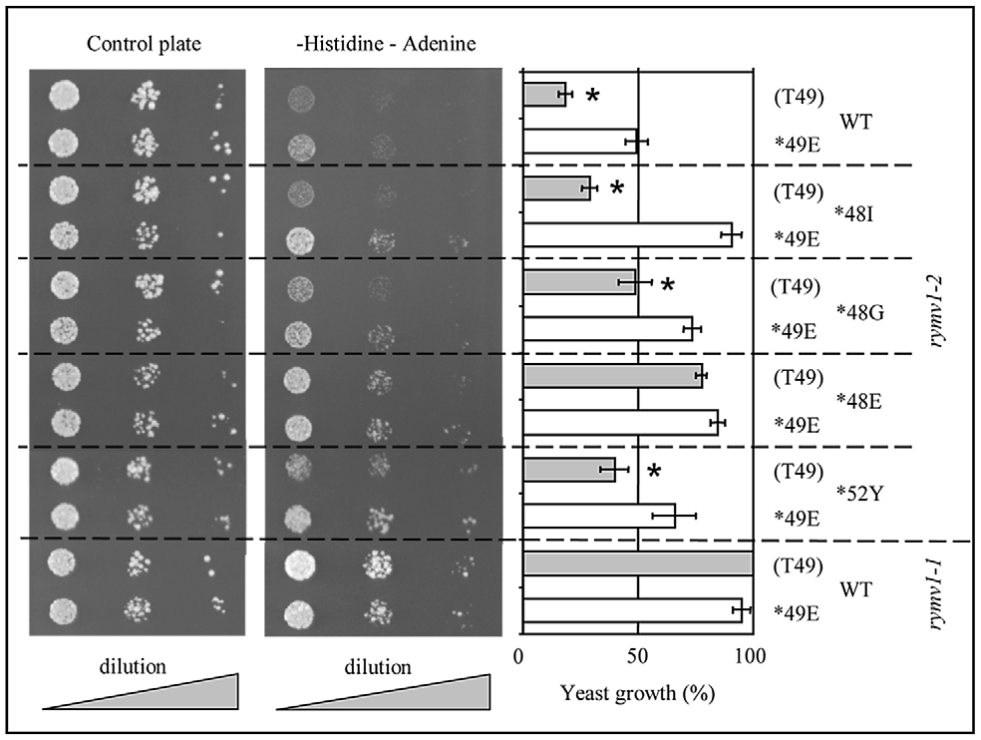
Two-hybrid assays of the interaction between *rymv1-2* mutated MIF4G and VPg with T49 or E49. Cells were plated as serial dilutions (optical density at 600 nm of 5×10–2, 5×10–3 and 5×10–4) on a control plate with (left panel) or without (middle panel) the histidine amino acid and the adenine base. Comparisons of yeast growth were estimated as percentages after quantifying spot intensities presented in the middle panel using ImageJ (fourth panel), with 100% control efficiency being assigned to the susceptible MIF4G (*rymv1-1*)/VPg (T49) binary interaction. Results were highly reproducible and are the means of four independent experiments. Significant differences in efficiency between isolates and mutants with T49 (grey bars) and E49 (white bars) at *P* = 0.05 after Student's test are indicated by a star.

Yeast double-hybrid assays were used to investigate the effect of the amino acid at codon 49 on the VPg/MIF4G interaction. The strength of the interaction between the MIF4G domain with the VPg of wild-type isolates (R48-H52), and of RB mutants *48I, *48G, *48E and *52Y with T49 or E49 was assessed. The mutated MIF4G domain mimicking the *rymv1-2* resistance substantially reduced the interaction with the wild-type VPg, whereas the VPg mimicking RB mutations partly or fully restored the interaction (Figure 5). These findings extended previous results with genotypes with T49 and RB mutations at codon 48 [17] to genotypes with E49 and RB mutations at codon 52. Overall, there was a marked effect of the amino acid at codon 49 on the strength of the interaction between the mutated MIF4G domain and the VPg of WT isolates and RB mutants. The interaction with the *rymv1-2* mutated MIF4G domain was significantly lower when VPg was carrying T49 than that observed in VPg with *49E. This was true both for the WT isolate (R48) and for RB mutants *48I, *48G and *52Y (*P*<0.001, *P*<0.001, *P*<0.025, respectively). The interaction was also lower in VPg with *48E-T49 than with *48E-*49E, although the difference was not statistically significant. Lower affinity to the *rymv1-* 2-MIF4G domain may contribute to the low propensity of isolates with T49 to overcome *rymv1-2* resistance. In particular, it may explain the inability of isolates with T49 to fix the *rymv1-2* RB mutation *52Y. Interaction between the VPg and the mutated MIF4G domain mimicking the *rymv1-3* resistance was different. There was no significant influence of the amino-acid at codon 49 on the strength of the interaction between the WT or *52Y mutated VPg and the *rymv1-3* mutated MIF4G (data not shown).

### Role of the amino acid at codon 49 in the adaptation to African and Asiatic rice species

We tested whether the amino acid at codon 49 of the VPg in wild-type isolates conferred a selective advantage in the infection of susceptible *O. glaberrima*, *O. sativa indica* and *O. sativa japonica* cultivars. Isolates representative of the diversity of RYMV were selected (cf. Materials and Methods). Preliminary experiments showed that single accumulation of each isolate, evaluated by qRT-PCR, was not significantly different between susceptible *O. sativa* and *O. glaberrima* cultivars (data not shown). Several pairs of isolates with E49 and T49 were then co-inoculated at the same concentration to *O. glaberrima*, *O. sativa indica* and *O. sativa japonica* cultivars. The outcome of the competition was assessed by sequencing the VPg of the viral population at successive dates after inoculation. Each co-inoculation was replicated at least twice. Altogether, the results of 92 competitions were sequenced during this study.

The outcome of the co-inoculation of T49 and E49 isolates in susceptible cultivars depended upon the rice species (Table 3). In *O. glaberrima* cultivars, the results were always clear-cut. Whatever the cultivar tested or the pair of isolates inoculated, the isolates with E49 were rapidly excluded (i.e. within 28 days) and only the isolates with T49 were fixed. The relationship between the amino acid at codon 49 and the result of the competition was highly significant in *O. glaberrima* (χ^2^ = 36, P<0.001). In *O. sativa indica* cultivars, the exclusion of one of the two genotypes also occurred but more slowly (i.e. within 60 days). Either isolates with E49 or T49 were fixed and there was no significant relationship between the amino acid at codon 49 and the outcome of the competitions. In *O. sativa japonica* cultivars, isolates with E49 or T49 were fixed, but there was a preferential fixation of isolates with T49 (χ^2^ = 9.8, *P* = 0.02). However, coexistence of the two genotypes was sometimes observed until 100 dpi.

**Table 3:** Outcome of the competitions between RYMV isolates with T49 and E49 after co-inoculation of susceptible *O. glaberrima*, *O. sativa indica* and *O. sativa japonica* cultivars.

Results^1^ of the competitions^2^	Rice species inoculated		
	*O. glaberrima* 3	*O. sativa indica* 4	*O. sativa japonica* 5
Dominance of isolates with T49	36	16	12
Dominance of isolates with E49	0	17	4
Co-dominance of isolates with T49 and with E49	0	0	7
Total number of competitions analysed	36	33	23

1 the outcome of the competition was assessed by analysis of the electrophoregrams.

2 CIa/CI4, CIa/Mg16, CIa/Tz209, BF1/Ma10, BF1/Tz8, BF5/CI4, BF5/Mg16, BF5/Tz209, Ni1/Ma10, Ni1/Tz8, CIa/CIa*49E; see text for details.

3 cvs. Tog5673, CG14, G39.

4 cvs. IR64, Bouaké189, BG90-2.

5 cvs. Nipponbare, Azucena.

The isolate CIa (T49) and the mutant CIa*49E were inoculated at the same concentration to susceptible *O. glaberrima*, *O. sativa indica* and *O. sativa japonica* cultivars to further assess the effect of the amino acid at codon 49 on the outcome of the competitions. Preliminary experiments showed there was no significant difference in virus accumulation after single inoculations (evaluated by DAS-ELISA and qRT-PCR) between the *O. sativa indica* cv. IR64 and the *O. glaberrima* cv. Tog5673 (Figure S1). The results of the competitions were consistent among the six replicates. In the *O. glaberrima* susceptible cv. Tog5673, the isolate CIa (T49) always displaced the single mutant CIa*49E. In the *O. sativa indica* and the *O. sativa japonica* susceptible cultivars, the single mutant CIa*49E displaced the isolate CIa (T49).

## Discussion

The first objective of this study was to establish that the amino acid at codon 49 of the VPg was not only a molecular ‘signature’ but was a key genetic determinant of the ability to overcome *rymv1-2* and *rymv1-3* resistance alleles. This was shown by directed mutagenesis of an infectious clone. The replacement of threonine by glutamic acid at codon 49 reversed the ability of isolate CIa to overcome *RYMV1* resistance alleles and modified the mutational pathways which were followed. Considering both its resistance breaking ability and the kind of RB mutations fixed, the mutant CIa*49E responded like the isolates with E49, and no longer like the isolate CIa (T49) or any other isolates with T49. This result validates the claim that the amino acid at codon 49 modulates the ability of RYMV isolates to overcome *rymv1-2* and *rymv1-3* resistances either by promoting or by restraining the fixation of RB mutations at virulence codons. Consistently, the virulence spectrum of *Potato virus Y* (PVY) on the *pvr2* resistance in pepper is dependent on epistatic interactions between nearby codons of the VPg [19]. Indeed, there is increasing phylogenetic and experimental evidence that epistatic relationships play a critical role in the adaptation of viruses (reviewed in [20]). This justifies our attempts to investigate the modes of action of the amino acid at codon 49 on the ability to break *RYMV1* resistance.

The second objective of this study was to determine the effects of the amino acid at codon 49 in the main *rymv1-2* and *rymv1-3* virulence pathways. The lack of fixation of the *rymv1-2* RB mutation *48G in genotypes with T49, and of the *rymv1-3* RB mutation *52Y in genotypes with E49 involved different processes. Genotypes with E49-*52Y were not infectious in *rymv1-3* resistant cultivars but were fit in susceptible and *rymv1-2* resistant cultivars. In contrast, genotypes with *48G-T49 were unfit in both susceptible and resistant cultivars. Combination *48W-T49 [15] in the clone CIa was also unfit in both susceptible and resistant cultivars. Actually, the range of possible changes within this motif is restricted, as indicated by the coordinated changes associated with the E49T substitution at codons 48-49-50 during RYMV evolution. Double-hybrid tests revealed that a threonine at codon 49 reduced the affinity of the RB mutants with the MIF4G domain of *rymv1-2*. The lower affinity to the MIF4G domain of *rymv1-2* possibly explain why mutation *52Y was not fixed after inoculation of isolates with T49. Altogether, the effects of the amino acid at codon 49 on *RYMV1* resistance breakdown found in this study are complex, critical, and numerous, and yet unlikely to be exhaustive.

Similarities in the response of *rymv1-3* and *rymv1-4 O. glaberrima* resistant cultivars to RYMV despite the differences of their genetic determinants (deletion vs. point mutation) suggested that *RYMV1* resistance breakdown was host-species dependent. Codon 49 of the VPg and codon 303 of MIF4G epitomized on the virus and plant sides, respectively, the effect of the genetic background on the outcome of the infection of *RYMV1* resistant cultivars. This was exemplified by mutation *52Y which was fixed in isolates with E49 in *rymv1-2* resistant *O. sativa* cultivars with A303. In constrast, mutation *52Y was fixed in isolates with T49 in *rymv1-3* resistant *O. glaberrima* cultivars with D303. Consistently, mutation *52Y was also fixed after inoculation of isolate with T49 to the *rymv1-4* resistant *O. glaberrima* cv. Tog5672 (A. Nicaise and A. Pinel-Galzi, unpublished results).

It was earlier stressed that the strategy of a RNA plant virus such as RYMV to gain virulence against host resistance showed striking parallels with HIV resistance against antiviral treatments [13]. The present finding supports this parallel. Epistatis within the 48-49-50 motif of the VPg is a major constraint to RYMV evolution by preventing key adaptive mutational pathways. Recently, it was also established that a major consequence of epistatis for HIV evolution is that many of the minimum-length mutational trajectories between the wild type and the mutant with highest fitness are selectively inaccessible [21].

The third objective of this study was to determine the origin of the amino acid polymorphism at codon 49. Altogether, a threonine at codon 49 conferred a strong selective advantage over glutamic acid in the *O. glaberrima* cultivars. Independent fixation events of T49 account for the diversifying selection estimated at codon 49 [13], [15]. The selective advantage of isolates with T49 in *O. glaberrima* over isolates with E49 is a likely explanation of these fixations. This is one of the first experimental validation of the adaptive role of a codon predicted to be under positive selection in the host range expansion of a pathogen in a natural environment [22]. The selective advantage of isolates with T49 in *O. glaberrima* also provides an explanation of the link between the threonine at codon 49 and the ability to overcome *rymv1-3* resistance in *O. glaberrima*. Isolates with T49, which are better adapted to *O. glaberrima* than isolates with E49, would be more prone to break *O. glaberrima rymv1-3* resistant cultivars. In contrast, isolates with E49, which are less fit in *O. glaberrima*, would not be able to overcome *rymv1-3* resistance. Consistently, the frequency of breakdown of an eIF4E resistance in pepper by mutations in the VPg of PVY was high in a susceptible genetic background and low when the same gene was introgressed in a partially resistant genetic background [23]. Results of the competitions between isolates with E49 and T49 in *O. sativa* were more variable than in *O. glaberrima*. The narrow genetic diversity of *O. glaberrima* [24]–[26] may explain the homogenous outcome of the competitions whereas the wide genetic diversity in *O. sativa* may account for the diversity in responses.

It has been proposed that a direct interaction between the eIF4E of pepper and the VPg of PVY drives the co-evolution between resistance and pathogenicity, leading to the diversifying selection of both genes [27], [28]. This is considered to be the best example of plant-virus co-evolution [29]. It was of interest to see whether this evolutionary scenario was relevant to RYMV in rice. Superficially, the evolution of eIF(iso)4G in rice and of the VPg in RYMV presents features similar to such an “arms race” co-evolution model. Under a co-evolution model similar to that between pepper and PVY, the resistance mutations in rice should have been fixed under the selection pressure imposed by RYMV. However, this hypothesis does not match what is known about the epidemiology of the virus. There is evidence that RYMV diversified only two centuries ago and that epidemics developed only in recent decades [30], [31]. This short time-scale is inconsistent with the hypothesis of rice adaptation to RYMV. More likely, the *RYMV1* resistance alleles resulted from past selection pressure imposed under a wider time-frame [30], possibly that of RYMV-related sobemoviruses [9]. RYMV variation does not fit a simple rice-RYMV co-evolution scenario either. With PVY, the codons of the VPg under diversifying selection are the virulence codons involved in eIF4E resistance breakdown [32], [33]. According to a co-evolution model, the virulence mutations were fixed under the selection pressure imposed by the resistance genes. With RYMV however, virulence codons of the VPg of wild type isolates are not variable, possibly because resistant cultivars have not yet been deployed widely in the fields. Most importantly, codon 49 - which is a under strong diversifying selection [13] - is not a virulence codon itself but is adjacent to virulence codons 48 and 52. Its indirect mode of action on resistance breakdown suggests an evolutionary scenario different from co-evolution.

In conclusion, our results – although superficially consistent with a co-evolution scenario – suggest a different historical relationship between RYMV and rice. On the host plant side, the *RYMV1* resistance alleles were presumably not fixed under the selection pressure of RYMV. On the virus side, the current ability to overcome resistances in the two rice species is modulated by past host adaptations and subsequent genetics constraints which are epitomized by a single amino acid of the VPg. This genetic determinant of *RYMV1* resistance breakdown exemplifies the importance of historical contingencies in the adaptability of a virus to a new host.

## Materials and Methods

### Plant material

Twelve rice cultivars were used in the experiments: three susceptible *O. sativa indica* cvs. (IR64, Bouaké189, BG90-2), three susceptible *O. glaberrima* cvs. (Tog5673, CG14, G39), two *O. sativa japonica* cvs. (Nipponbare, Azucena), two *rymv1-2* resistant *O. sativa indica* cvs. (Bekarosaka, Gigante), the *rymv1-3* resistant *O. glaberrima* cv. Tog5681 and the *rymv1-4* resistant *O. glaberrima* cv. Tog5672. In addition, two NILs were constructed by introgression of the *rymv1-3* and *rymv1-2* resistance alleles into the susceptible *O. sativa indica* cv. IR64. In the *rymv1-3* NIL, the estimated percentage of *O. glaberrima* genes introgressed in the *O. sativa indica* cultivar was below 10%. Moreover, mapping this isogenic line showed that *O. glaberrima* genes potentially involved in the viral cycle such as translation initiation factor genes were not introgressed in the susceptible *O. sativa indica* cultivar (data not shown). Then, differences between the *rymv1-2* and *rymv1-3* NILs were mostly restricted to the *RYMV1* gene. The plants were kept in a growth chamber under 12 hours illumination at 120µE-2s-1 at 28°C and 90% humidity.

### Viral isolates, mutagenesis of infectious clone and inoculation

Our experiments followed a two-step approach: (i) directed mutagenesis of the VPg of the infectious clone CIa (T49), (ii) comparison of a range of field isolates with E49 and T49. The mutants CIa*49E, CIa*48G, CIa*48G*49E, CIa*52Y (T49) and CIa*49E*52Y were constructed by directed mutagenesis of the infectious clone CIa (T49) with QuikChange Site-Directed Mutagenesis Kit (*Stratagene*) [13], [15]. Transcription of mutated clones and inoculation *in planta* of viral RNAs was done as described elsewhere [34]. None of the results obtained suggested a loss of competitive fitness of the mutated infectious clone CIa*49E. Nine wild-type isolates, including isolates of the three clades with T49, were selected as representative of the diversity of the virus to be used in the experiments (Table S1).

These isolates were from Burkina-Faso (BF1, BF5), Côte d'Ivoire (CIa, CI4), Madagascar (Mg16), Mali (Ma10), Nigeria (Ni1) and Tanzania (Tz8, Tz209). Isolates CIa (strain S2-S3), BF1 (strain S2-S3), BF5 (strain S1-wa) and Ni1 (strain S1-c) had a threonine at codon 49 of the VPg. Isolates CI4 (S1-wa), Ma10 (strain S1-wa), Mg16 (strain S4-mg), Tz8 (strain S4-lm) and Tz209 (strain S6) had a glutamic acid at codon 49. The genotype Mg16*52Y (E49) was obtained after the inoculation of the *rymv1-2* resistant plants [13], [15]. The RB genotypes Ma105*52Y (T49) and Ma203*Y52 (T49) were obtained after the inoculation of the *rymv1-3* resistant plants. The isolates were multiplied in the *O. sativa* susceptible cv. IR64 and were stored in a -20°C deep freezer before use in the experiments. The VPg of the 11 field isolates was sequenced as described in [13]. There were few differences between the amino acid sequences and none of them correlated with the polymorphism at position 49 (Figure S2). Interestingly, the amino-acid sequence of the VPg of the mutant CIa*49E fully matched that of the field isolate Tz209. No mutation of T49 towards E49 (or vice versa) in the VPg was observed at the multiplication stages or after inoculation of susceptible or resistant *O. sativa* or *O. glaberrima* cultivars.

Inoculum was prepared by grinding infected frozen leaves in 0.1 M phosphate buffer (0.1 g/ml, pH 7.2) and was rubbed on leaves of 14-day-old rice seedlings. Viral quantification was performed by q-RT-PCR as described previously [14]. For competition experiments, ca. 10^12^ copies of each isolate were mixed and inoculated to the different cultivars. In parallel, the same amount of inoculum of each isolate was inoculated singly to each cultivar. To test the RB ability of CIa and CIa*49E genotypes, ca. 10^13^ copies of each genotype were inoculated to resistant hosts. The accumulation of mutants CIa*48G, CIa*48G*49E, CIa*52Y and CIa*49E*52Y was compared after the inoculation of ca. 10^12^ copies of each genotype per plant. All genotype sequences were checked before and after inoculation (see below).

### Viral quantification, sequence analysis and two-hybrid assays

For each competition experiment in susceptible plants, expanded leaves of different plants were collected at 14, 21, 28, 56 and 100 dpi. The VPg was then sequenced by analyzing RT-PCR fragments amplified from total RNA purified by RNeasy Plant Mini Kit (*QIagen*) as previously described [35]. Dual peaks in the sequencing electropherograms at the same position were interpreted as reflecting the co-existence of the two genotypes in the viral population [13], [36]. In constrast, a single peak at a site polymorphic between the two competing genotypes suggested that one genotype predominated. The ELISA tests were performed on the leaf samples as described previously [37].

Two hybrid assays were conducted with the Matchmaker GAL4 Two-Hybrid System 3 (Clontech). The yeast strain AH109 was co-transformed with either pGAD::MIF4G (*rymv1-1*) or pGAD::MIF4G* (mimicking the *rymv1-2* allele) and either pGBK::VPg (wild-type with R48-H52) or pGBK::VPg* (mimicking RB mutants *48I, *48G, *48E and *52Y) constructs. Double-transformation of yeast cells and spotting on control or selective medium plates was performed as previously reported [17]. Yeast cells were grown at 30°C for 4 days. Growth intensity was monitored with ImageJ software [38]. The data were normalized to positive and negative controls and expressed as a percentage after quantifying spot intensities, assigning 100% efficiency to the susceptible *rymv1-1* MIF4G/wild-type VPg interaction. At least four independent experiments were conducted. The results of the virus accumulation estimated by DAS-ELISA, and of the interaction efficiency evaluated in yeast two hybrid systems were analysed by ANOVA (Statistica software version 6.0).

## Supporting Information

Figure S1 Virus accumulation of the isolate CIa (T49) (white bars) and of the mutant CIa*49E (grey bars) in the susceptible *O. sativa indica* cv. IR64 and in the susceptible *O. glaberrima* cv. Tog5673 assessed by DAS-ELISA (absorbance at 405 nm). The vertical bars show the standard deviation of the mean calculated from assessment of three plants.
(TIF)

Figure S2 Amino acid diversity of the VPg of wild type isolates. The amino acid sequence of the isolate CIa - used to construct the CIa infectious clone - is given in the top row. The amino acid at position 49 of the VPg of each isolate is indicated in plain letter. The differences between the VPg sequences of the wild type isolates with that of CIa isolate are indicated in plain letters.
(TIF)

Table S1 Name, strain and isolation host of the isolates and mutants used, and accession numbers of their VPg.
(XLS)

## References

[1] Kouassi N, N'Guessan P, Albar L, Fauquet C, Brugidou C (2005). Distribution and characterization of *Rice yellow mottle virus*: A threat to African farmers.. Plant Dis.

[2] Allarangaye M, Traoré O, Traoré E, Millogo R, Guinko S (2007). Host range of rice yellow mottle virus in Sudano-Sahelian Savannahs.. Pakist J Biol Sciences.

[3] Portères R, Fage J, Oliver R (1970). Primary cradles of agriculture in the African continent.. Papers in African Prehistory.

[4] Linares O (2002). African rice (*Oryza glaberrima*): History and future potential.. Proc Natl Acad Sci USA.

[5] Vaughan D, Lu B, Tomooka N (2008). The evolving story of rice evolution.. Plant Science.

[6] Li Z, Zheng X, Ge S (2011). Genetic diversity and domestication history of African rice (*Oryza glaberrima*) as inferred from multiple gene sequences.. Theor Appl Genet.

[7] Ndjiondjop M, Brugidou C, Zang S, Fargette D, Ghesquière A (2001). High resistance to *Rice yellow mottle virus* (RYMV) in two cultivated rice cultivars is correlated to the failure of cell-to-cell movement.. Physiol Mol Plant Pathol.

[8] Albar L, Bangratz-Reyser M, Hébrard E, Ndjiondjop MN, Jones M (2006). Mutations in the eIF(iso)4G translation initiation factor confer high resistance of rice to *Rice yellow mottle virus*.. Plant J.

[9] Thiémélé D, Boisnard A, Ndjiondjop M, Chéron S, Séré Y (2010). Identification of a second major resistance gene to *Rice yellow mottle virus*, *RYMV2*, in the African cultivated rice species, *O. glaberrima*.. Theor Appl Genet.

[10] Rakotomalala M, Pinel-Galzi A, Albar L, Ghesquière A, Rabenantoandro Y (2008). Resistance to *Rice yellow mottle virus* in rice germplasm in Madagascar.. Europ J Plant Pathol.

[11] Thottappilly G, Rossel H (1993). Evaluation of resistance to *Rice yellow mottle virus* in *Oryza species*.. Indian J Virol.

[12] Hébrard E, Pinel-Galzi A, Fargette D (2008). Virulence domain of the RYMV genome-linked viral protein VPg towards rice *rymv1–2*-mediated resistance.. Arch Virol.

[13] Pinel-Galzi A, Rakotomalala M, Sangu E, Sorho F, Kanyeka Z (2007). Theme and variations in the evolutionary pathways to virulence of an RNA plant virus species.. PLoS Pathog.

[14] Poulicard N, Pinel-Galzi A, Hébrard E, Fargette D (2010). Why *Rice yellow mottle virus*, a rapidly evolving RNA plant virus, is not efficient at breaking *rymv1-2* resistance.. Mol Plant Pathol.

[15] Traore O, Pinel-Galzi A, Issaka S, Poulicard N, Aribi J (2010). The adaptation of *Rice yellow mottle virus* to the eIF(iso)4G-mediated rice resistance.. Virology.

[16] Hébrard E, Bessin Y, Michon T, Longhi S, Uversky V (2009). Intrinsic disorder in Viral Proteins Genome-linked : experimental and predictive analyses.. Virology J.

[17] Hébrard E, Poulicard N, Gérard C, Traoré O, Wu H (2010). Direct interaction between the *Rice yellow mottle virus* VPg and the central domain of the rice eIF(iso)4G1 factor correlates with rice susceptibility and RYMV virulence.. Mol Plant Microbe Interac.

[18] Shapiro B, Rambaut A, Pybus OG, Holmes EC (2006). A phylogenetic method for detecting positive epistasis in gene sequences and its application to RNA virus evolution.. Mol Biol Evol.

[19] Ayme V, Petit-Piere J, Souche S, Palloix A, Moury B (2007). Molecular dissection of the potato virus Y VPg virulence factor reveals complex adaptations to the *pvr2* resistance allelic series in pepper.. J Gen Virol.

[20] Holmes EC (2009). The Evolution and Emergence of RNA Viruses..

[21] da Silva J, Coetzer M, Nedellec R, Pastore C, Mosier D (2010). Fitness epistasis and constraints on adaptation in a human immunodeficiency virus type 1 protein region.. Genetics.

[22] Moury B, Simon V (2011). dN/dS-based methods detect positive selection linked to trade-offs between different fitness traits in the coat protein of *Potato virus Y*.. Mol Biol Evol.

[23] Palloix A, Ayme V, Moury B (2009). Durability of plant major resistance genes to pathogens depends on the genetic background, experimental evidence and consequences for breeding strategies.. New Phytol.

[24] Second G (1982). Origin of the genic diversity of cultivated rice (*Oryza spp*). Study of the polymorphism scored at 40 isozyme loci.. Japan J Genet.

[25] Wang Z, Second G, Tanksley S (1992). Polymorphism and phylogenetic relationships among species in the genus *Oryza* as determined by analysis of nuclear RFLPs..

[26] Ishii T, Xu Y, McCouch S (2001). Nuclear- and chloroplast-microsatellite variation in A-genome species of rice.. Genome.

[27] Cavatorta J, Savage A, Yeam I, Gray S, Jahn M (2008). Positive Darwinian selection at single amino acid sites conferring plant virus resistance.. J Mol Evol.

[28] Charron C, Nicolaï M, Gallois J, Robaglia C, Moury B (2008). Natural variation and functional analyses provide evidence for co-evolution between plant eIF4E and potyviral VPg.. Plant J.

[29] Fraile A, Garcia-Arenal F (2010). The coevolution of plants and viruses: resistance and pathogenicity.. Adv Virus Res.

[30] Fargette D, Pinel-Galzi A, Sérémé D, Lacombe S, Hébrard E (2008). Diversification of *Rice yellow mottle virus* and related viruses spans the history of agriculture from the Neolithic to the present.. PLoS Pathog.

[31] Traore O, Pinel-Galzi A, Sorho F, Sarra S, Rakotomalala M (2009). A reassessment of the epidemiology of *Rice yellow mottle virus* following recent advances in field and molecular studies.. Virus Res.

[32] Moury B, Morel C, Johansen E, Guilbaud L, Souche S (2004). Mutations in *Potato virus Y* genome-linked protein determine virulence toward recessive resistances in *Capsicum annuum* and *Lycopersicon hirsutum*.. Mol Plant Microbe Interac.

[33] Ayme V, Souche S, Caranta C, Jacquemond M, Chadoeuf J (2006). Different mutations in the genome-linked protein VPg of *Potato virus Y* confer virulence on the pvr2^3^ resistance in pepper.. Mol Plant Microbe Interac.

[34] Brugidou C, Holt C, Yassi A, Zhang S, Beachy R (1995). Synthesis of an infectious full-length cDNA clone of rice yellow mottle virus and mutagenesis of the coat protein.. Virology.

[35] Hébrard E, Pinel-Galzi A, Bersoult A, Siré C, Fargette D (2006). Emergence of a resistance-breaking isolate of *Rice yellow mottle virus* during serial inoculations is due to a single substitution in the genome-linked viral protein VPg.. J Gen Virol.

[36] Poon A, Kosakovsky Pond S, Bennett P, Richman D, Brown A (2007). Adaptation to human populations is revealed by within-host polymorphisms in HIV-1 and Hepatitis C virus.. PLoS Pathog.

[37] N'Guessan P, Pinel A, Sy A, Ghesquière A, Fargette D (2001). Distribution, pathogenicity, and interactions of two strains of *Rice yellow mottle virus* in forested and savanna zones of West Africa.. Plant Dis.

[38] Abramoff M, Magelhaes P, Ram S (2004). Image processing with ImageJ.. Biophotonics Internat.

