# Supplementary figures and images for: Historical Contingencies Modulate the Adaptability of Rice Yellow Mottle Virus
Source: PLoS Pathog. 2012 Jan 26;8(1):e1002482. doi: 10.1371/journal.ppat.1002482 (PMC3266926; doi:10.1371/journal.ppat.1002482)

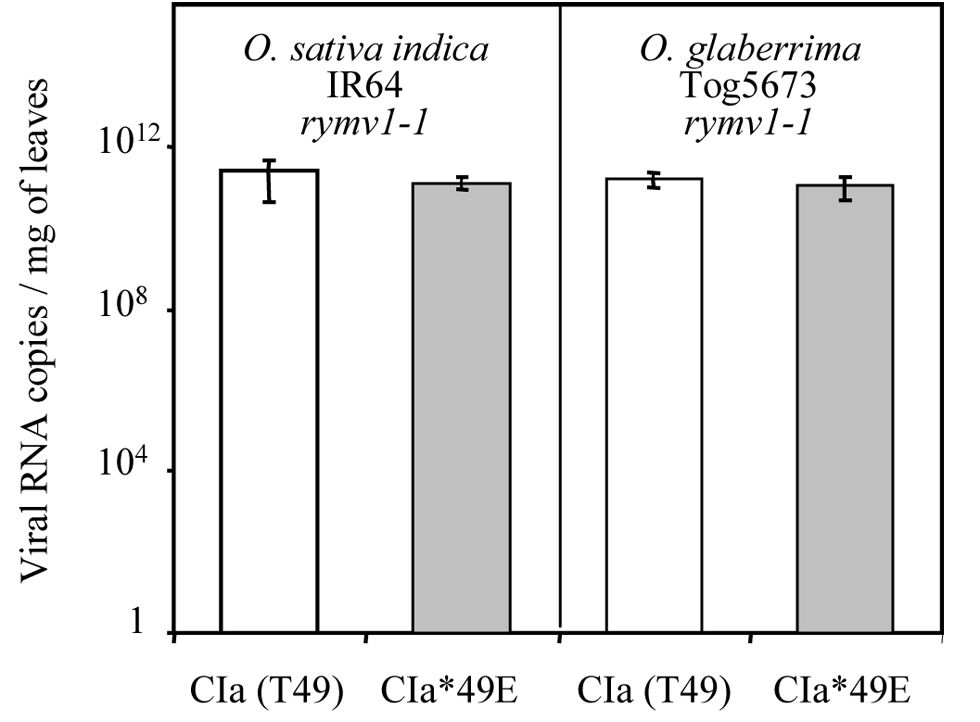

Supplement: Figure S1 — Virus accumulation of the isolate CIa (T49) (white bars) and of the mutant CIa*49E (grey bars) in the susceptible O. sativa indica cv. IR64 and in the susceptible O. glaberrima cv. Tog5673 assessed by DAS-ELISA (absorbance at 405 nm). The vertical bars show the standard deviation of the mean calculated from assessment of three plants. (TIF) [file ppat.1002482.s001.tif]

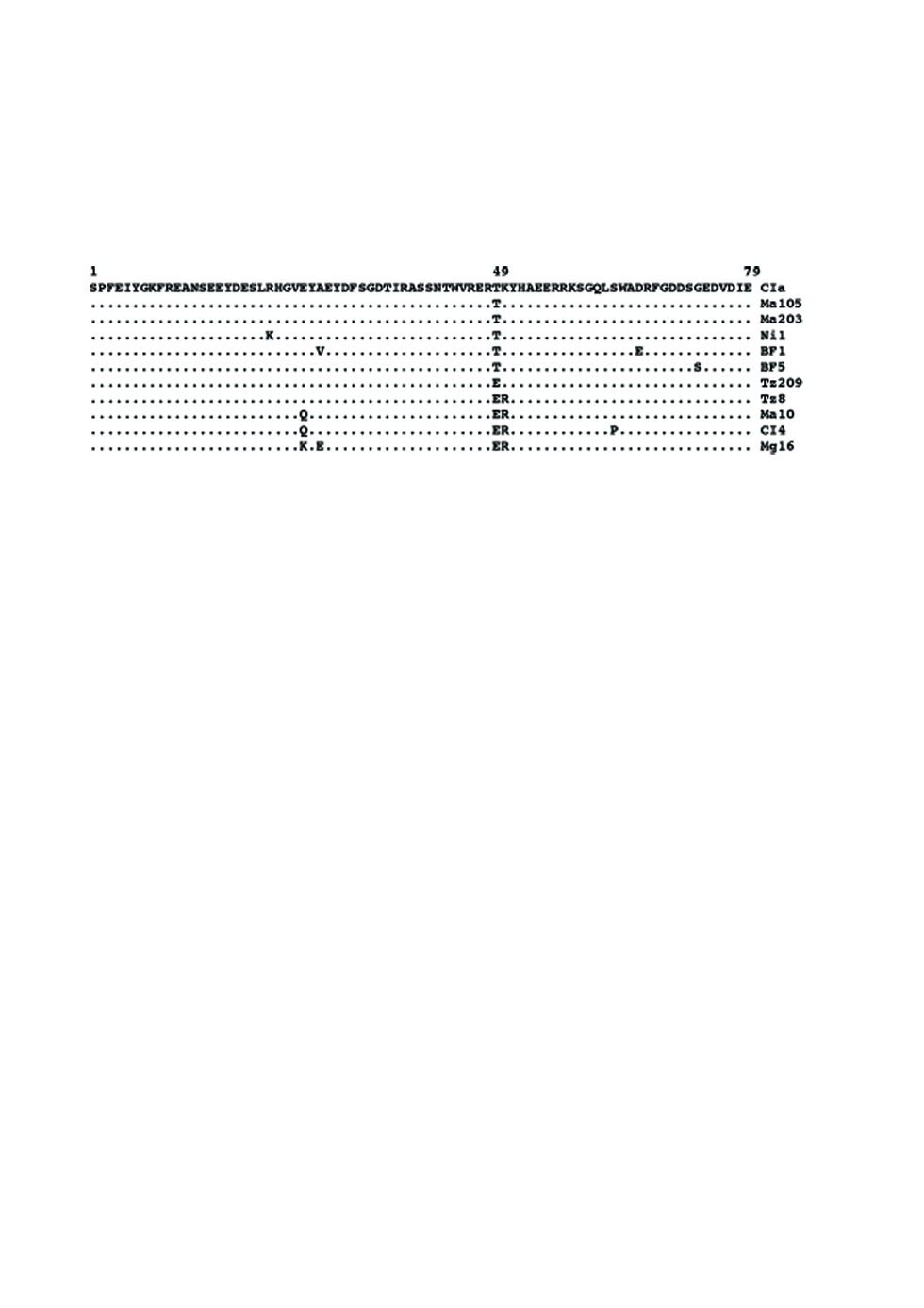

Supplement: Figure S2 — Amino acid diversity of the VPg of wild type isolates. The amino acid sequence of the isolate CIa - used to construct the CIa infectious clone - is given in the top row. The amino acid at position 49 of the VPg of each isolate is indicated in plain letter. The differences between the VPg sequences of the wild type isolates with that of CIa isolate are indicated in plain letters. (TIF) [file ppat.1002482.s002.tif]
